# A Concise History of the Principal Discoveries in Anatomy

**Published:** 1799-08

**Authors:** 


### *A concise History of the principal Discoveries in Anatomy.*

[Extracted from the Original German, of Prof. CURT. SPRENGEL'S "*History of Medicine*," Vol. III. Sect. XI. p. 503, and fol.]

§. 1. NO century has ever been so productive of great and interesting discoveries; in none has the knowledge of the human body increased so rapidly; and there never was a time in which so great a number of the most enlightened minds were emulous to improve anatomy, that highly important and necessary branch of human knowledge, as during the 16th century, the period of which we here treat.

Convinced of the importance of that department, Prof. SPRENGEL avows that he has, for several years, devoted a principal share of his attention to this this branch of medical hiſtory; that he has, during that time, purſued the moſt induſtrious ſtudy of the anatomical writers of the the ſixteenth century; and hence he concludes, that this ſection of his work cannot fail to be highly intereſting to the ſcientific reader. But in order to combine brevity with a perſpicuous arrangement, the learned Profeſſor previouſly gives the literary accounts of the moſt celebrated anatomiſts of the ſixteenth century, and afterwards ſtates their different diſcoveries, in ſcientific order.

§. 2. Among the anatomiſts of that century, *Veſalius* is perhaps the moſt illuſtrious, or at leaſt the moſt celebrated, and unqueſtionably the firſt who powerfully oppoſed inveterate prejudices, as well as that implicit adherence to *Galen*, while he diſcovered, and, without heſitation, expoſed the errors of the Greek author. Hence originated an important epocha in medical ſcience; and it will appear in the courſe of this inquiry, from various individual inſtances, that the influence of his reformation, on his contemporaries, as well as the ſubſequent writers, was conſiderable and permanent. The anatomiſts who lived previous to his time, made indeed ſeveral valuable diſcoveries, and partly deſcribed nature as ſhe really is, without imitating the erroneous repreſentation of Galen, but they ſtill conſidered it as an unwarrantable boldneſs, to contradict that great, and, in their opinion, incomparable writer. Under ſuch unfavourable circumſtances, this ſcience could make but little progreſs, till the immortal Veſalius appeared, who ſucceſsfully broke the fetters of prejudice, and above all things earneſtly recommended the ſtudy of nature.

*Gabriel Zerbi* is the earlieſt anatomiſt of the ſixteenth century, and his work "*Anatomia corporis humani:* fol. Venet. 1502," is written throughout in the manner of *Mondini*, inſomuch that it is ſcarcely conceivable, how the pre-eminent performance of Veſalius could appear in the ſhort interval of forty years after that inelegant work. Zerbi was a native of Verona, and filled the academical chair for ſome time at Verona, and afterwards at Rome. Having been guilty of a criminal action, he was obliged to fly to the Turkiſh dominions, where he met with an unfortunate cataſtrophe. Having performed a palliative cure on a baſhaw, he was purſued, apprehended, and cut to pieces by the ſervants of the deſpot.

*Alexander Achilini* was attached as much as Zerbi to the method and the prejudices of Mondini, while he combined with them an intolerable ſcholaſtic verboſity. He was profeſſor at Bologna, and is known in medical hiſtory by his diſputes with *Pompanazzi*, in which he profeſſed the principles of *Averrhoës*. His book, entitled "*Achillini annotationes anatomicæ in Mundinum:*" fol. Bonon. 1522, contains, however, a variety of intereſting obſervations, together with ample proofs that the author has diligently diſſected diſſected human bodies. The ſame remark applies to *Nicolaus Maſſa* whoſe work "*Anatomiæ liber introductorius*," 4to. Venet. 1559, is not deſtitute of original obſervations, while it likewiſe affords inſtances to prove the author's adherence to the prevailing prejudices of the age.

Nor is *Joh. Winter of Andernach*, an author of great merit, and he has according to the teſtimony of his pupil, Veſalius*, little, if at all, applied himſelf to the ſtudy of nature. In his work entitled "*Anatomicarum Inſtitutionum*," lib. iv. 8vo. Baſil. 1536, ſome diſcoveries are recorded, which he certainly has not made.

*Andr. Laguna*, of whom we have already made honourable mention in the fifth ſect. of the preceding Hiſtory of Surgery, is the author of an anatomical manual, entitled "*Andr. Lacunæ anatomica methodus*," 8vo. Paris. 1535, a work written in a ſtrange metaphorical ſtyle, yet not entirely void of original facts and remarks.

§. 3. *Jacob Berengar de Carpi* deſerves to be recorded as a worthy predeceſſor of Veſalius. He was Profeſſor at Bologna, from the year 1502 to 1527; and it is related of him, as a remarkable fact, that he gave the firſt anatomical demonſtrations on the body of a hog, in the houſe of Albert Pio, Lord of Carpi, and that he had ſubſequently diſſected upwards of one hundred human ſubjects. He ſtands alſo charged with having performed diſſections on living human bodies; a charge which the vulgar are apt to bring againſt every zealous anatomiſt. His great and numerous diſcoveries juſtly obtained the diſtinguiſhed approbation of *Fallopia*, one of the moſt competent judges, who appreciated their value in his "*Obſervationes Anatomicæ*," p. 365.

*Jacob du Bois*, or *Sylvius*, the teacher of Veſalius, has likewiſe made many important diſcoveries, although he was involved in violent diſputes with his pupil; by ſome authors (*Riolan. anthropogr.* lib. i. c. 5. p. 29,) he is conſidered as the firſt reſtorer of anatomy in France, becauſe he diſſected human bodies inſtead of thoſe hogs, in order to give his anatomical demonſtrations from the former. He was probably the inventor of injections, as he firſt mentions them in his "*Iſagoge Anatomica*," p. 66. But his great predilection for the ancient writers miſled him to the moſt palpable errors. In general, he was an accurate obſerver; but whatever Galen had differently deſcribed, he conſidered as a deviation from the natural ſtate, and frequently had recourſe to the abſurd idea, that human nature was degenerated, and that therefore many things were found different from what they were when Galen

* "Tot mihi modo ſectiones infligi cupio, quod illum in homine aut in alio bruto (præterquam in menſa) tentantem vidi."—*Veſal. de radic. chyn. epiſt.* p. 675.

Galen deſcribed them. Of his injuſtice to Veſalius we ſhall find in the progreſs of this inquiry ſeveral remarkable inſtances.

§. 4. The great and comprehenſive mind of *Andreas Veſalius*, whoſe name no friend of anatomy will pronounce without veneration, is an ornament of the German nation, although he was born at Bruſſels. He ſtudied in Löwen and Paris, under Sylvius, where he frequently, with no ſmall danger, gratified his inſatiable deſire of anatomizing. He afterwards ſerved as phyſician in the Imperial army. but ſoon retired to Italy, and began, firſt at Padua, to teach anatomy with uncommon reputation, ſo that he had, at times, five hundred pupils. He alſo reſided at Bologna and Piſa, till he publiſhed his large and immortal work, in conſequence of which, he was called to the court of the Emperor Charles V.—He was likewiſe appointed firſt phyſician to Philip II. his ſon and ſucceſſor to the imperial crown; and among other ſucceſsful cures, he healed Don Carlos of a dangerous wound in the head. At length, Veſalius travelled into Paleſtine, and died on his return, after having been ſhipwrecked on the iſland of Zanthe.

The greateſt merit of Veſalius indiſputably conſiſts in his criticiſms on Galen's aſſertions; and although he has been cenſured for having ſometimes purpoſely miſ-interpreted the text, he has nevertheleſs, in moſt inſtances, ſucceſsfully diſcloſed the errors of Galen, and ſhown how inconſiſtently former anatomiſts had proceeded, in blindly following the precepts of that author. It were much to be wiſhed, however, that the reproaches with which he loads the Greek phyſician, for having placed too much reliance on the diſſections of animals, might not be equally applicable to Veſalius; for we ſhall find in the ſequel, that his obſervations are frequently liable to ſimilar objections.

Veſalius acquired another great advantage over his predeceſſors, by having, with the aſſiſtance of the celebrated artiſts, *Tizian*, and *John of Calkar*, furniſhed the firſt accurate and finiſhed anatomical repreſentations drawn from nature. Yet he found frequent cauſe of complaint againſt thoſe artiſts, becauſe the delineation of the parts of the human body had not for them ſufficient intereſt. It has been ſaid, that his were the firſt faithful copies made from nature; for the drawings which *Leonardo da Vinci* had executed for *Marc. Ant. della Torre*, were not publiſhed, but ſcattered after the death of the latter. Nor have the plates been preſerved which the immortal *Michael Angelo Buonarotti*, a great adept in anatomy, had engraved with his own hand.

§. 5.

§. 5. The attention which the work of Vesalius excited, was conformable to the expectations formed of this magnificent performance. The succeeding anatomists now endeavoured either to defend the authority and supposed infallibility of Galen—they proceeded on the road of discovery pointed out by Vesalius—or they were blind imitators and copyists of what he and his great predecessors had stated in their works.

Among the most zealous partisans of Galen's anatomical system, we find principally *Franc. Puteus*, of Vercelli, who published, in 1562, his "*Apologia pro Galeno*, 8vo. Venet." in which he studiously and earnestly endeavours to prove, that Galen had actually dissected human bodies. He at the same time expresses the singular wish, that no discoveries whatever might be published, which tend to diminish the authority of his beloved ancients; but that all such pretended discoveries should be deposited in a public edifice, as was the case in the temple at Cos, in order to ascertain their respective merits, by an accurate and impartial investigation *. He is probably not always in the wrong, when he, in a variety of instances, disputes the accuracy of representation in the plates of Vesalius; yet the blame in such cases attaches more justly to the artist than to the author. Vesalius defended himself against *Puteus*, under the assumed name of *Gabr. Cuneus*; but this apology has not obtained the approbation of impartial judges, because this great writer too frequently repeats his arguments in that essay. He likewise complains of the enmity he experienced from *Joh. Dryander*, Professor at Marburg, who made Mondini's Manual the text-book in his anatomical lectures, and was a faithful follower of this inferior writer. Dryander began his academical instructions in 1535, since which period public dissections have been performed in that university. The representations of the parts of the human body which he annexed to this work, entitled "*Anatomiæ pars prior.*" 4to. Marb. 1537, are as rough and unfinished as the drawings published in the work of *Lewis Levasseur (Vassæus)*, of Chalons-sur-Marne, whose compendium consists chiefly of extracts from the writings of Galen.

*Charles Etienne*, of the celebrated family of the *Stephani*, and who was himself the director of a printing-office, and professor of anatomy at Paris, has

* If all *modern* professors of anatomy were compelled to submit their discoveries and improvements to public scrutiny, before they be permitted to claim their share of merit in new or *renewed* discoveries,—it would be an excellent means of preventing many unnecessary disputes, as well as useless verbosity, in their lectures and performances; and the time thus wasted might be beneficially employed in *more luminous* demonstration. W.

has indeed made many useful discoveries, and several interesting observations; but his adherence to Galen frequently prevented him from discovering the truth, and he was consequently unacquainted with various particulars ascertained prior to his time. The drawings given in his work, under the title "*Stephanus de dissectione partium corp. hum.*" Fol. Paris. 1546, were vindicated in a French treatise on the origin and progress of Surgery, by *Stephen de la Riviere.*

§. 6. *Bartholom. Eustachius,* of Sanseverino near Salerno, Professor in Rome, and first Physician to Cardinal d' Urbino, whimsically combined the most profound anatomical knowledge with the most implicit adherence to the principles of Galen. It is but too obvious from his writings, how frequently his submission to Galen revolted against reason and experience, which, it is to be regretted, were but too often slighted. But notwithstanding this unfavourable circumstance, Eustachius has acquired great and permanent merit by uniting the comparative anatomy of animals with that of the human species; a subject which has been carried to a high degree of perfection in his excellent writings, called "*Eustachii Opuscula.*" 8vo. Lugd. Bat. 1707. His plates "*De renum structura,*" c. 16. p. 44. which were engraved during his life time, in the year 1552, but were considered as lost for a century and a half, till the Pope at length made a present of them to his physician, *Lancisi,* who first published them at Rome, in 1714, are lasting monuments of his anatomical knowledge and skill. They were subsequently republished at Rome, in 1740, by a surgeon named *Cajetan Petrioli,* who edited them with a jargon of unintelligible "*Riflessioni*;"—and soon afterwards the world was presented with the excellent and classical edition of these plates, published by *Albinus,* at Leyden, in 1744 and 1761; and the Commentaries by *Martin,* printed at Edinburgh, 8vo. 1740.—The intention of Eustachius, in the elaborate publication of his anatomical tables, has been very accurately ascertained both by Martin, and the great physiologist, Haller, namely that he did not wish to represent all the parts of the human body in their natural order, but had purposely exhibited his own delineations in such a manner as to enable anatomists to correct by them the assertions of Vesalius, and at the same time to represent the discoveries peculiar to Eustachius in a more conspicuous light. These tables frequently afford the best documents for deciding the controversies then especially prevailing; and it is remarkable, as has already been noticed by Albinus, that most of the drawings are taken from young subjects. But there is contained in them an inexhaustible stock of new discoveries and observations, the most important of which will be hereafter recorded.

§. 7. Several

§. 7. Several anatomiſts learnt from Veſalius the free method of inveſtigating old prejudices, and conſequently again endeavoured to improve upon, and to ſupply the deficiencies in the obſervations made by this great writer, whether in point of accuracy in the deſcription of parts, or in the preciſion of the concomitant explanations; many of his ſucceſſors, however, did not treat him with the deference due to his talents, as they wiſhed to acquire reputation by undervaluing his merits: while others obſerved in their writings that degree of delicacy which the greatneſs of his mind, as well as the integrity of his intentions juſtly demanded, and conſequently rectified his errors, and improved upon his ideas, with modeſt and philoſophic reſerve. Among the latter we may principally place *Joh. Bapt. Cannani*, Profeſſor in Ferrara.

It is much to be regretted that, of his work on the muſcles, an outline only has been tranſmitted to poſterity, the drawings of which were executed by the celebrated artiſt, *Hieron. Carpenſis*. Of all medical works this is perhaps the ſcarceſt; and Prof. Sprengel availed himſelf of the copy extant in the Electoral library at Dreſden,* from which he has extracted the moſt important obſervations; as will appear in the ſequel of the preſent inquiry.

Another author, named *Joh. Phil. Ingraſſias*, in a work entitled "*Ingraſſias in Galeni libr. de oſſibus commentar.*" fol. Panorm. 1603, improved upon the diſcoveries of Veſalius in oſteology, and treated on the bones of the human body with an accuracy and attention to the ſmalleſt minutiæ, which affords almoſt complete ſatisfaction.—But the egoiſtic *Realdus Columbus*, of Cremona, a pupil of Veſalius whom he ſucceeded in the academical chair, at Padua, though he afterwards reſided at Rome, did not treat his great maſter with becoming deference. His work "*De re anatomica*," lib. xv. 8vo. Francof., 1593, is a teſtimony of his unbounded egotiſm, a deſire of innovation, and neglect of ſimple truth; while it muſt be allowed that he has made many valuable diſcoveries, and that, from his great experience, having diſſected ſometimes fourteen ſubjects in the courſe of

---

* This work is entitled: "Muſculorum humani corporis picturata diſſectio, per *Job. Bapt. Cannanum*, Ferrarienſem Medicum, in Barthol. Nigrifoli, Ferrar. patricii, gratiam nunc primum in lucem edita eſt."—There is no year nor place of printing mentioned in the title-page; the whole is comprised in a few ſheets text, and twenty-ſeven plates in Quarto; and on the title-page are written the words: *Sum Andreæ Aurifabri Vratis lav. Doct.* 1545. *Venetiis.*"—Beſides *Haller*, no modern anatomiſt or medical hiſtorian has inſpected this work; and it is ſuppoſed, that there exiſt only three copies of it.—Prof. Sprengel publicly acknowledges his obligations to the Aulic Counſellor *Adelung*, the great German philologiſt, and Firſt Librarian at Dreſden, by whoſe liberality he obtained the loan of that ſcarce book.

of one year, he was eminently qualified to become the commentator of Galen and Veſalius.—In the diſſections of living animals, he firſt made uſe of dogs in preference to hogs, which were uſually employed by his predeceſſors.

A man of a different character, and more ſplendid talents than either Veſalius or Euſtachius, was *Gabriel Fallopia*, who combined the moſt profound erudition with the moſt amiable modeſty and impartiality; he was thoroughly acquainted with the ſtructure of the human body; his ſtyle is manly and didactic, equally remote from verboſity and obſcurity; in ſhort, he was a man whoſe illuſtrious example has been productive of ſo many beneficial conſequences, that our hiſtorian, Profeſſor Sprengel, on account of all theſe ſuperior qualifications, is inclined to pronounce Fallopia the firſt anatomiſt of the ſixteenth century. He was a native of Modena; had ſtudied in Padua, under Veſalius; afterwards obtained a prebendary in Modena, from whence he went upon extenſive travels into France and Greece, and ſucceſſively filled the anatomical chairs of Ferrara, Piſa, and Padua. From a paſſage in his work, entitled, "*De tumore præternatur.*" c. xiv. p. 632. it appears, that the anatomiſts of thoſe times, when they were in want of ſubjects, applied to the government of the country, with a requeſt to obtain the bodies of criminals, whom, in ſuch caſes, the anatomiſts deſtroyed, as Fallopia ſays, *in their own way*, that is, by a proper doſe of *opium*, and then undertook the diſſection.

§. 8. The individual diſcoveries by which *Jul. Cæſar Aranzi* is known in the anatomical world, we ſhall have occaſion to point out more minutely in the ſequel: he wrote a work, entitled, "*Arantius de humano fœtu cum obſervationibus*," 4to. Venet. 1595. He as well as *Conſtantin Varoli*, Profeſſor at Bologna, and phyſician to the Pope, inveſtigated more minutely the diſcoveries of *Veſalius*, and has furniſhed us with many uſeful remarks. His work, entitled, "*De nervis opticis epiſtola*;" 8vo. Patav. 1573, contains abundant proofs of this aſſertion; and he was the firſt who has, more minutely than his predeceſſors, examined the formation of the brain, together with the inſertions of the nerves.—In like manner did *Joh. Bapt. Carcano Leone*, Profeſſor at Pavia, endeavour to correct the occaſional miſtakes committed by Veſalius and Fallopia, in his Treatiſe, "*Anatomicæ*," *libr.* ii. 8vo. Ticini, 1574, where he particularly complains, that anatomiſts too frequently attempt to apply the reſults drawn from the diſſections of animals, to the appearances noticed in the human body.

The name of *Volcher Koyter*, of Groeningen, deſerves an honourable place in the hiſtory of anatomy, on account of his work publiſhed in

1574, at Nürnberg, under the title "*Coiteri externarum et internarum corporis humani partium tabulæ atque anatomicæ exercitationes observationes-que variæ.*" He had studied under Fallopia, Eustachius, Rondelet, and Aldrovandi; prosecuted his anatomical inquiries for several years, at Nürnberg, where he cultivated comparative anatomy with uncommon ardour and success, after having been previously engaged as physician in the war against the French, and has transmitted to posterity many excellent and valuable observations on particular parts of the human body. With similar regard, we must mention *Salomon Alberti*, of Naumberg, Professor at Wittenberg, who is known by an useful Compendium, entitled, "*Historia plerarumque partium humani corporis*," 8vo. Witteb. 1601; a work which is not destitute of interesting observations.

Lastly, *Hieronymus Fabricius*, of Aquapendente, deserves to be recorded in the list of eminent anatomical observers. He was a worthy pupil and successor of Fallopia, whose great example he followed, in explaining, from the comparative anatomy of man and animals, the functions of the human body. In his excellent work, entitled, "*Fabricii opera omnia anatomica*," fol. Lips. 1687, he has presented us with many important discoveries.

§. 9. Among the less eminent promoters of anatomical study, or those who are chiefly reputed as compilers and imitators, *Job. Valverde de Hamusco* deserves the first place; he wrote a work on the anatomy of the human body, in the Spanish language; which was printed in 1560, folio, at Rome, where it was likewise translated into Italian. This large book is, with a few exceptions, chiefly to be considered as an extract from the writings of Vesalius.

An anatomical compendium, not unlike the last mentioned, was published by *Guido Guidi*, a native of Florence, who caused the plates of Vesalius to be re-engraved, and described the parts throughout in conformity to this model. Similar works have been transmitted to us by *Felix Plater* *, and *Caspar Bauhin* †, Professors at Basle. The latter justly claims the additional merit of having collected all synonymous anatomical terms, and invented new and appropriate names for the parts discovered in the human body: by this useful work, much confusion has been prevented which would have been inevitable, if one anatomist had continued to call a muscle the first, which another called the second. Bauhin, however,

* *De partium corporis humani structura et usu.* fol. Basil. 1583.

† *Institutiones anatomicæ.* 8vo. Basil. 1592.—*Ejusd.* Theatrum anatomicum. 4to. Francof. 1621.

ever, has made no peculiar discoveries; nay, he even claimed *Varoli's* cuts representing the brain, as his original design, though he had not the least title to their relative merit.

*Joh. Posthius*, of Gemmersheim, in the Palatinate, a pupil of *Rondelet* and *Joubert*, and afterwards physician to the Bishop of Würzburg, and the Elector Palatine, published some supplements to the Manual of Columbus, which have been annexed to the edition of this work published by Professor Sprengel.

Two other, but very indifferent anatomical writers of this century should be mentioned, namely, *Archangelo Piccolhuomini* *, of Ferrara, and *Andr. Du Laurens* †, of Arles. The former was Professor at Rome, and lost his reputation by neglecting the discoveries of his predecessors, making consequently many erroneous observations, giving incorrect drawings of the parts he had observed, and introducing much confusion in anatomical science. Du Laurens was an extremely unaccomplished man; yet, notwithstanding his ignorance, he was made Chancellor of the University of Montpellier ‡, first physician to the court of France, and Dean of the Medical Faculty at Paris. His work below quoted, is a compound of superstitious, misconceived, and ill-applied principles, while it bears striking evidence, that he has not made proper use of the great discoveries then extant, of his predecessors as well as his contemporaries.

[*In our next Number we shall resume this historical sketch, and communicate the most important discoveries themselves, in systematic order.*]

---

* *Piccolhuomini anatomicæ prælectiones.* fol. Rom. 1586.

† *Laurentii historia anatomica.* 8vo. Francof. 1602.

‡ *Primirose*, in his Treatise, " *De vulgi erroribus*," lib. i. c. 2. p. 4, relates of him, that the University above-mentioned would not acknowledge him as their chancellor, till he had obtained the immatriculation, and defended as many public theses as were prescribed by the various degrees necessary to the appointment to that elevated situation.

